# KIF2C Deletion Causes Meiotic Abnormalities and Nonobstructive Azoospermia in Mice

**DOI:** 10.1002/rmb2.12659

**Published:** 2025-05-27

**Authors:** Hiroaki Kitakaze, Haruhiko Miyata, Yuki Oyama, Chen Pan, Yuma Kujime, Go Tsujimura, Takahiro Imanaka, Sohei Kuribayashi, Norichika Ueda, Kentaro Takezawa, Shinichiro Fukuhara, Norio Nonomura, Masahito Ikawa

**Affiliations:** ^1^ Research Institute for Microbial Diseases The University of Osaka Osaka Japan; ^2^ Department of Urology, Graduate School of Medicine The University of Osaka Osaka Japan; ^3^ Graduate School of Pharmaceutical Sciences The University of Osaka Osaka Japan; ^4^ The Institute of Medical Science The University of Tokyo Tokyo Japan; ^5^ Center for Infectious Disease Education and Research The University of Osaka Osaka Japan; ^6^ Center for Advanced Modalities and DDS (CAMaD) The University of Osaka Osaka Japan

**Keywords:** KIF2C, knockout mice, male infertility, nonobstructive azoospermia

## Abstract

**Purpose:**

Kinesin Family Member 2C (KIF2C) is a key regulator of microtubule dynamics and chromosome segregation in mitosis. However, its role in spermatogenesis remains unclear. Recent transcriptomic analyses suggest a potential link between KIF2C and male infertility. This study aimed to clarify KIF2C's roles in spermatogenesis using *Kif2c* knockout (KO) mice.

**Methods:**

To overcome the preweaning lethality associated with *Kif2c* deletion, we generated *Kif2c* KO mice with a mixed genetic background of 129X1/SvJ and B6D2. We assessed male fertility, epididymal sperm counts, and testicular sections of *Kif2c* KO mice.

**Results:**

Global *Kif2c* KO mice were obtained and showed male infertility. Histological analyses and epididymal sperm count revealed that *Kif2c* KO mice exhibited severely impaired spermatogenesis and absence of mature spermatozoa. These findings are consistent with those observed in patients with nonobstructive azoospermia (NOA). Our classification of *Kif2c* KO seminiferous tubules indicated that most spermatogenic cells were arrested at the early stages, particularly during meiosis.

**Conclusions:**

This study provides in vivo evidence that KIF2C is essential for spermatogenesis and male fertility in mice. The successful generation of global *Kif2c* KO mice establishes an animal model for NOA, supporting research on germ cell development and reproductive health.

## 1 Introduction

**FIGURE 1:**
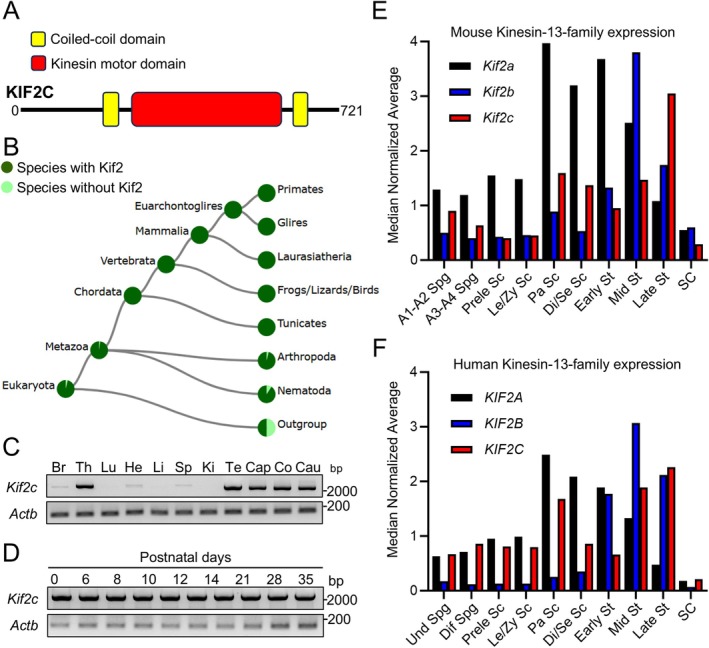
*Kif2c* is evolutionarily conserved and expressed in testes. (A) Mouse KIF2C has two coiled‐coil domains and a kinesin motor, catalytic domain. (B) Conservation of *Kif2* among different species. (C) RT‐PCR analysis of *Kif2c* expression in various mouse tissues. *Actb* was used as a loading control. Br: Brain, Th: Thymus, Lu: Lung, He: Heart, Li: Liver, Sp: Spleen, Ki: Kidney, Te: Testis, Cap: Caput epididymis Co: Corpus epididymis, Cau: Cauda epididymis. (D) RT‐PCR analysis of *Kif2c* expression in various mouse postnatal testes. *Actb* was used as a loading control. (E) Expression of mouse kinesin‐13‐family genes in testes was analyzed using single‐cell transcriptome data. A1‐A2 Spg: A1 and A2 differentiating spermatogonia, A3‐A4 Spg: A3 and A4 differentiating spermatogonia, Prele Sc: Preleptotene spermatocytes, Le/Zy Sc: Leptotene/zygotene spermatocytes, Pa Sc: Pachytene spermatocytes, Di/Se Sc: Diplotene/secondary spermatocytes, Early St: Early round spermatids, Mid St: Mid round spermatids, Late St: Late round spermatids, SC: Sertoli cells. (F) Expression of human kinesin‐13‐family genes in testes was analyzed using single‐cell transcriptome data. Und Spg: Undifferentiating spermatogonia, Dif Spg: Differentiating spermatogonia.

Kinesin is a motor protein involved in various cellular processes [1]. Kinesin Family Member 2C (KIF2C), also known as Mitotic Centromere‐Associated Kinesin (MCAK), is part of the kinesin‐13 family of proteins. In contrast to conventional kinesins, which have the motor domain in the N‐terminus and transport cargos along microtubules, kinesin‐13 family proteins contain the motor domain in the middle of the molecules (Figure 1A) and have the function of depolymerizing microtubules [2, 3]. It has been shown that KIF2C plays a critical role in microtubule dynamics and chromosome segregation in mitosis [4, 5]. Aberrant expression of KIF2C has been observed in various cancers, including breast, lung, gastric, and prostate tumors [6, 7, 8, 9]. In addition, overexpression of KIF2C could lead to chromosomal aberrations that promote tumor progression [10]. Conversely, inhibition of KIF2C has been shown to suppress tumor cell proliferation, suggesting that it may serve as a potential therapeutic target in cancer treatments [11, 12]. While KIF2C has been extensively studied in the context of mitosis and oncogenesis, its roles in meiosis and spermatogenesis are poorly understood.

Several studies have suggested a potential association between KIF2C and male infertility. Analyses of Gene Expression Omnibus (GEO) datasets from patients with nonobstructive azoospermia show that *KIF2C* is significantly downregulated in spermatogenesis [13]. Additionally, transcriptomic and single‐cell RNA sequencing analyses have identified KIF2C as one of four novel hub genes linked to Klinefelter syndrome [14], a major cause of azoospermia and oligospermia due to impaired spermatogenesis [15, 16]. It has also been shown that KIF2C is localized to the inner centromere during meiosis in mouse spermatocytes [17]. These findings suggest that KIF2C may be a crucial regulatory gene in male fertility and could serve as a potential biomarker or therapeutic target for male infertility.

Very recently, KIF2C was reported to be essential for male fertility using mice with conditional knockout (cKO) of *Kif2c* in germ cells [18]; however, it is unclear if global *Kif2c* KO mice show similar spermatogenetic phenotypes. Although cKO models enable tissue‐specific functional analyses, recombination efficiency is not always perfect, which could complicate the interpretation of phenotypes [19, 20]. According to the International Mouse Phenotyping Consortium (IMPC), *Kif2c* KO mice show preweaning lethality [21, 22], making it challenging to analyze KIF2C functions using a global KO mouse model. To overcome this limitation, we generated *Kif2c* KO mice with a mixed genetic background of B6D2 and 129X1/SvJ, and obtained sexually mature mice for analyzing their fertility phenotypes.

## 2 Materials and Methods

### 2.1 Animals

All animal experiments were approved by the Animal Care and Use Committee of the Research Institute for Microbial Diseases, The University of Osaka (approval number: #Biken‐AP‐H30‐01 and #Biken‐AP‐R03‐01). Mice were purchased from CLEA Japan (Tokyo, Japan) or Japan SLC (Shizuoka, Japan). Wild‐type (WT) or *Kif2c* heterozygous (HET) mice were used as controls. Frozen spermatozoa of *Kif2c* HET mice generated in this study will be made available at the RIKEN BioResource Research Center (Ibaraki, Japan) and the Center for Animal Resources and Development, Kumamoto University (Kumamoto, Japan).

### 2.2 RT‐PCR

RNA was extracted from multiple adult tissues and postnatal testes of C57BL/6N mice using TRIzol (Thermo Fisher Scientific, MA, USA) according to the manufacturer's protocol. The obtained RNA was reverse transcribed to cDNA with a SuperScript III first‐strand synthesis system (Thermo Fisher Scientific). PCR of *Kif2c* and *Actb* was performed using 10 ng of cDNA with KOD Fx Neo DNA Polymerase (Toyobo, Tokyo, Japan) and primers listed in Table S1. The amplification conditions for the PCR were 35 s at 94°C, followed by 30 cycles of 98°C for 10 s, 60°C for 30 s, and 72°C for 2 min 30 s, with a final 3 min extension at 72°C.

### 2.3 In Silico Analyses

The domain of mouse KIF2C was analyzed using the Simple Modular Architecture Research Tool (SMART) (http://smart.embl‐heidelberg.de/). Single‐cell transcriptome data of Kinesin‐13‐family in the mouse and human testes were obtained from the previously reported database [23]. Kinesin‐13‐family expression was analyzed using the Loupe Browser 7 (10× Genomics, Pleasanton, CA, USA). To compare the amino acid sequences of human KIF2C and mouse KIF2A, 2B, and 2C, BLAST (https://blast.ncbi.nlm.nih.gov/Blast.cgi), Clustal Omega [24], and Jalview [25] were used.

### 2.4 Generation of *Kif2c* Knockout (KO) Mice

*Kif2c* KO mice were generated using the CRISPR/Cas9 system as described previously [26]. Female B6D2F1 mice were super‐ovulated by intraperitoneal injection of CARD HyperOva (Kyudo, Saga, Japan) and human chorionic gonadotropin (hCG) (ASKA Pharmaceutica, Tokyo, Japan). Subsequently, female mice were caged with WT B6D2F1 males. The resulting two‐pronuclear (2PN) zygotes were isolated from the female mice. The sequences of two gRNAs were listed in Table S1, which target exon 1 and exon 20 of *Kif2c*, respectively. 2PN zygotes were electroporated by super electroporator NEAP21 (NEPA GENE, Chiba, Japan) with the crRNA/tracrRNA/Cas9 ribonucleoproteins (tracrRNA: #TRACRRNA05N‐5NMOL, Sigma‐Aldrich, MO, USA, and CAS9 protein: #A36497, Thermo Fisher Scientific). Treated zygotes were cultured and developed into 2‐cell stage embryos in potassium simplex optimized medium (KSOM) [27], and were transplanted into the oviducts of pseudopregnant ICR female mice the next day. Pups were either naturally delivered or obtained through Cesarean section and genotyped by PCR with the primers listed in Table S1 and subsequent Sanger sequencing of the PCR products.

### 2.5 Mating Tests

Sexually matured *Kif2c* WT and KO male mice were housed with three 8‐week‐old B6D2F1 female mice for 3 months, and vaginal plugs and the number of pups were checked and recorded every morning during the first 2 months. Only the number of pups was recorded for the rest of the month.

### 2.6 Sperm Count per Epididymis

All spermatozoa collected from the cauda epididymis were suspended in a 100 μL drop of TYH medium [28]. After incubation for 10 min at 37°C under 5% CO_2_, the spermatozoa were diluted with water, and the sperm count per epididymis was calculated using a hemocytometer.

### 2.7 Periodic Acid‐Schiff (PAS) Staining

PAS staining was performed as previously described [29]. Testes or epididymis were fixed at 4°C in Bouin's solution (Polysciences Inc., Warrington, PA, USA) and processed for paraffin embedding. Paraffin sections were cut at a thickness of 5 μm using an HM325 microtome (Microm, Walldorf, Germany). After rehydrating the sections, they were stained with 1% periodic acid (Nacalai Tesque, Kyoto, Japan) and Schiff's reagent (FUJIFILM WakoPure Chemical, Osaka, Japan) for 20 min each at room temperature. The sections were then counterstained with Mayer's hematoxylin solution (FUJIFILM WakoPure Chemical). The sections were observed with an Olympus BX‐53 microscope (Tokyo, Japan).

### 2.8 TUNEL Staining

Apoptotic cells in seminiferous tubules were detected using the in situ apoptosis detection kit (Takara Bio Inc., Shiga, Japan), which is designed to detect fragmented DNA histochemically by TUNEL [Terminal deoxynucleotidyl Transferase (TdT)‐mediated dUTP Nick End Labeling]. Testes were fixed at 4°C in Bouin's solution (Polysciences Inc.) and processed for paraffin embedding. The paraffin sections were cut to a thickness of 5 μm, and after rehydration, the sections were incubated for 20 min in 10 mM citric acid buffer at pH 6.0 heated to 95°C. To inactivate endogenous peroxidase activity, the sections were incubated in 3% H_2_O_2_ at room temperature for 5 min. After washing three times with PBS, the sections were incubated with TdT enzyme and FITC‐conjugated dUTP at 37°C for 1 h, and then washed with PBS. Next, the sections were incubated with HRP‐conjugated anti‐FITC antibody at 37°C for 30 min, and incubated with ImmPACT DAB substrate (Vector Laboratories, Burlingame, CA, USA) for 2–5 min. After washing with distilled water, the sections were stained with Mayer's hematoxylin solution for 3 min and observed with an Olympus BX‐53 microscope (Tokyo, Japan).

### 2.9 Statistical Analysis

All statistical analyses were performed using GraphPad Prism 10 (GraphPad Software, San Diego, CA, USA). Data are presented as the mean ± standard deviation (SD) unless otherwise stated. Differences between two groups were analyzed using Student's *t*‐test. For comparisons among three or more groups, one‐way ANOVA followed by Dunnett's multiple comparison test was performed. Statistical significance was defined as follows: ns (not significant), *p* < 0.05 (*), *p* < 0.01 (**), and *p* < 0.001 (***).

## 3 Results

### 3.1 *Kif2c* Is Evolutionarily Conserved and Expressed in Testes

Mouse KIF2C contains two coiled‐coil domains and a kinesin motor domain (Figure 1A), and is evolutionarily conserved in animals with high homology of amino acid sequences between mice and humans (Figure 1B and Figure S1). To evaluate the tissue expression of mouse *Kif2c*, we conducted reverse transcription polymerase chain reaction (RT‐PCR) using the primers listed in Table S1. Our results showed that *Kif2c* is highly expressed in the testis, epididymis, and thymus, while there is also a slight expression detected in the brain, heart, and spleen (Figure 1C). Furthermore, we conducted RT‐PCR on mouse testes at various postnatal days to determine when *Kif2c* begins to express during the first wave of spermatogenesis and found that *Kif2c* is expressed even at birth (postnatal Day 0) (Figure 1D). Consistently, the single‐cell transcriptome database [21] indicates that *Kif2c* is expressed from the early stage of mouse spermatogenesis (Figure 1E). The same database shows that *KIF2C* is also widely expressed from spermatogonia to spermatids in human spermatogenesis (Figure 1F). In mice and humans, there are three KIF2 proteins, KIF2A‐C. A BLAST search (https://blast.ncbi.nlm.nih.gov/Blast.cgi) shows that the amino acid sequence of mouse KIF2C is similar to that of mouse KIF2A (53% identity) and KIF2B (50% identity). *Kif2b*/*KIF2B* is highly expressed in spermatids compared to spermatogonia and spermatocytes, while *Kif2a*/*KIF2A* is widely expressed from spermatogonia to spermatids like *Kif2c*/*KIF2C* (Figure 1E,F).

### 3.2 *Kif2c* Deletion Leads to Preweaning Lethality in Mice With the B6D2 Genetic Background, but Crossing With the 129X1/SvJ Strain Mitigates the Lethality

**FIGURE 2:**
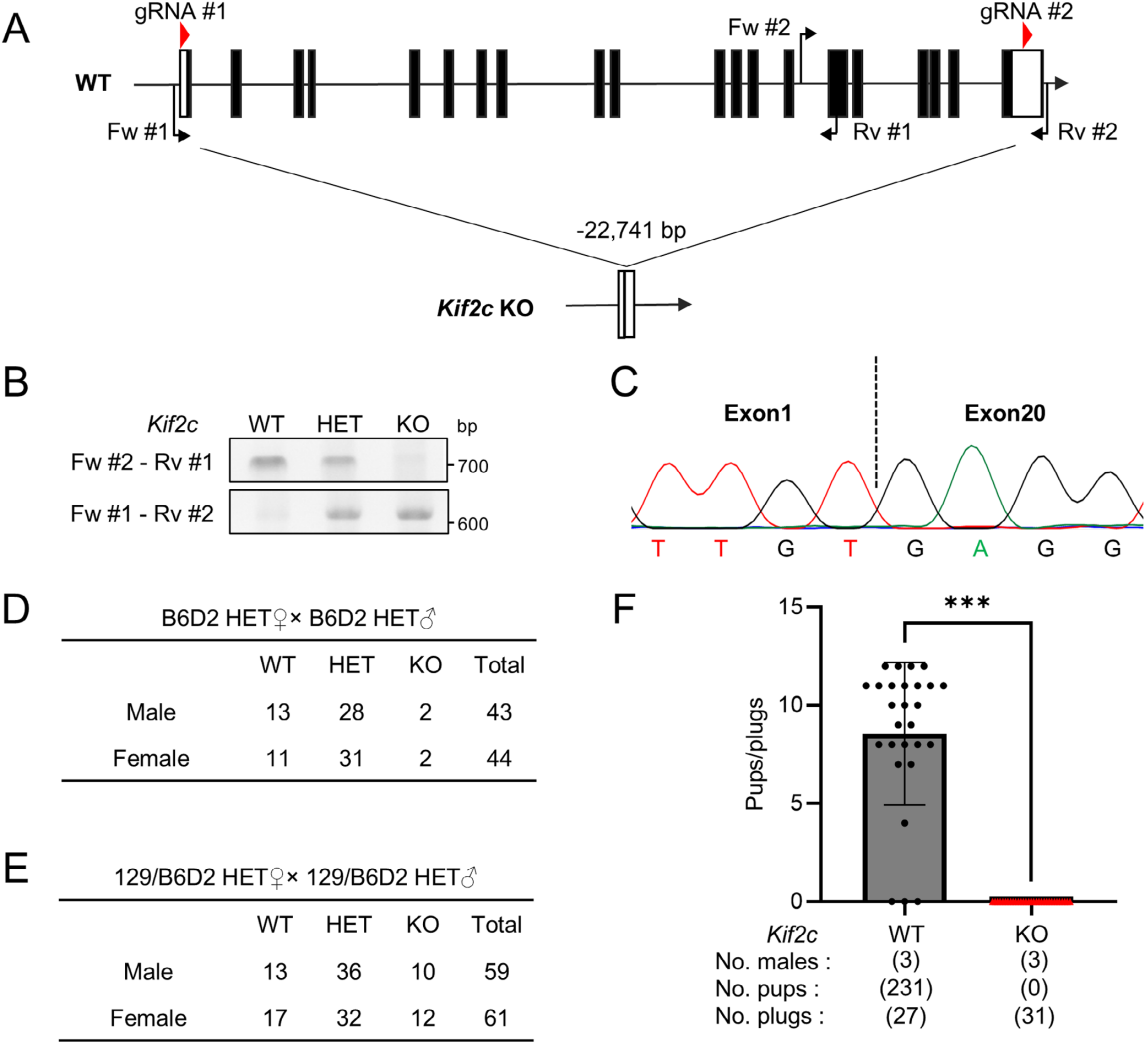
Deletion of *Kif2c* results in male infertility in mice. (A) A strategy for generating *Kif2c* KO mice using CRISPR/Cas9. The gRNA#1 and gRNA#2 were designed to target exon1 and exon 20, respectively, resulting in the deletion of 22,741 bp. Genotyping primers are labeled Fw #1–2 and Rv #1–2. Black rectangle: Translated region, white rectangle: Untranslated region. (B) Genotyping of *Kif2c* WT, HET, KO mice. Fw #2‐Rv #1 (for WT) and Fw #1‐Rv #2 (for KO) primers shown in (A) were used. (C) A truncation of 22,741 bp was detected in the *Kif2c* KO allele by Sanger sequencing. (D) The number of pups born from crosses of B6D2 *Kif2c* HET mice. (E) The number of pups born from crosses of 129/B6D2 *Kif2c* HET mice. (F) The number of pups born per plug. ***p < 0.001, Student's *t*‐test.

To explore the function of *Kif2c* in mouse spermatogenesis, we generated knockout (KO) mice using the CRISPR/Cas9 system to delete exons 1 to 20 of *Kif2c* in B6D2 mice (Figure 2A). We electroporated 70 zygotes obtained by B6D2F1 x B6D2F1 matings and transplanted 60 embryos into the oviducts of pseudopregnant females. We then obtained eight pups with the confirmation of a large deletion in two pups. These mice were genotyped with genomic PCR using primers shown in Figure 2A and Table S1 (Figure 2B) and subsequent Sanger sequencing of the PCR product (Figure 2C). F0 mice were mated with B6D2F1 WT mice to obtain *Kif2c* heterozygous (HET) mice. We then crossed B6D2 *Kif2c* HET mice and obtained a total of 87 pups; however, only 4 of these pups were homozygous for *Kif2c* KO when genotyped within 1 week after birth (Figure 2D), which is lower than the expected number according to the Mendelian ratio. Additionally, four *Kif2c* homozygous KO mice died within 10 weeks of birth. These results indicate that *Kif2c* KO mice with the B6D2 genetic background show preweaning lethality, which is consistent with the IMPC database.

Previous reports have shown that crossing mice with another strain can be an effective strategy to avoid lethality when generating KO mice [30, 31, 32]. In this study, we crossed B6D2 *Kif2c* HET male mice with 129X1/SvJ WT female mice to generate *Kif2c* HET mice with the mixed genetic background (129/B6D2). We then crossed 129/B6D2 *Kif2c* HET mice and obtained 22 homozygous *Kif2c* KO mice out of 120 pups (Figure 2E). Although the number is less than the expected number (30 pups) according to the Mendelian ratio, KO mice were sexually mature with no obvious abnormalities in appearance or behavior, and subjected to further analyses.

### 3.3 *Kif2c* Is Indispensable for Male Fertility in Mice

To investigate the effect of *Kif2c* deletion on male fertility, we performed mating tests of *Kif2c* WT or KO male mice with WT females. The average pup number per plug was 8.6 in *Kif2c* WT males, while no pups were obtained from *Kif2c* KO males despite observing 31 plugs (Figure 2F). These results indicate that *Kif2c* is essential for male fertility in mice.

### 3.4 Deletion of *Kif2c* Causes Nonobstructive Azoospermia With Hypospermatogenesis

**FIGURE 3:**
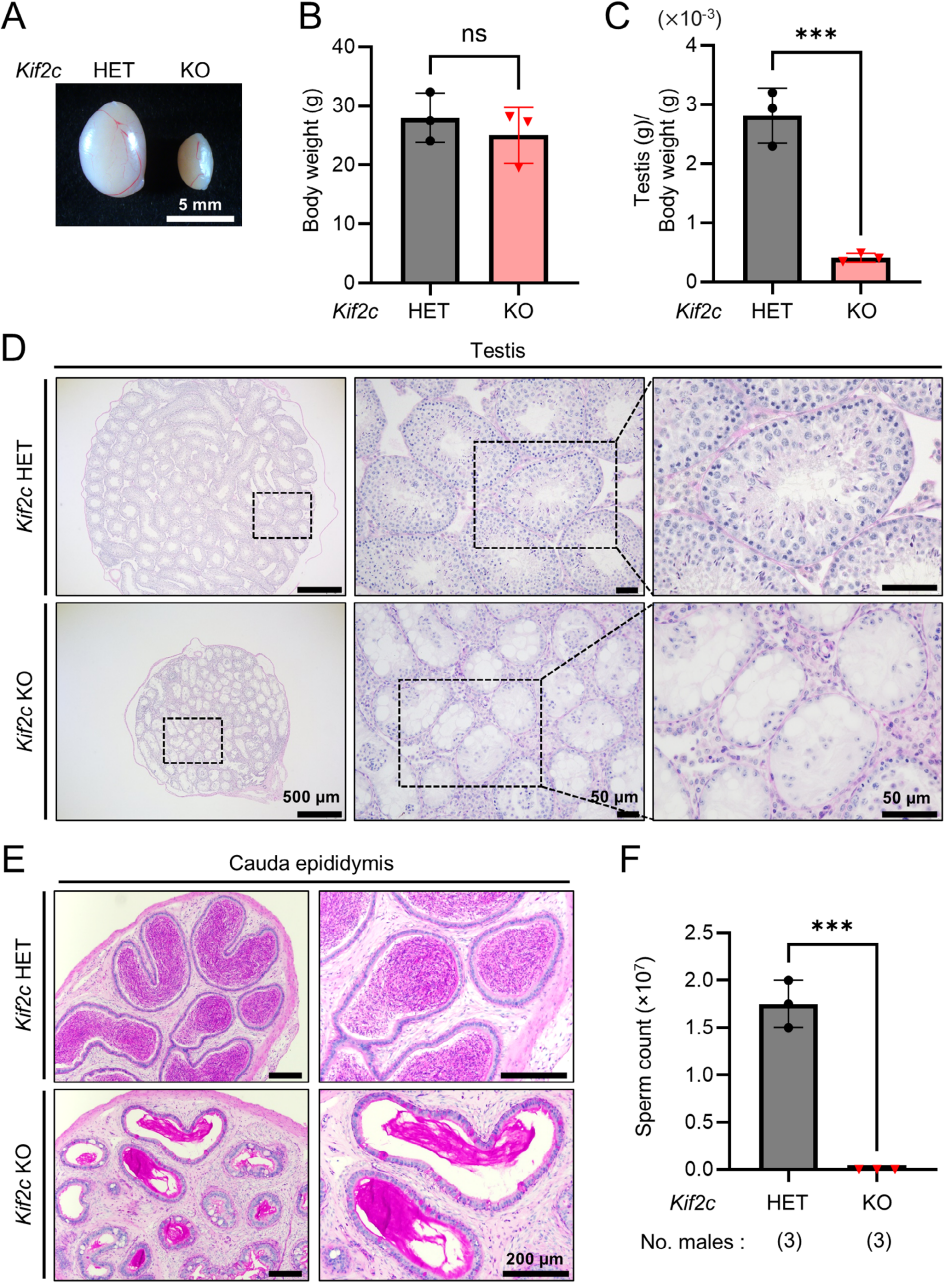
*Kif2c* KO mice show nonobstructive azoospermia with hypospermatogenesis. (A) Gross appearance of testes from adult *Kif2c* HET and KO mice. (B) Average body weight of adult *Kif2c* HET and KO mice. *n* = 3 males, *p* = 0.8208, Student's *t*‐test. (C) Average testis weight/body weight of adult *Kif2c* HET and KO mice. *n* = 3 males, ***p < 0.001, Student's *t*‐test. (D) PAS staining of testicular sections of adult *Kif2c* HET and KO mice. (E) PAS staining of the cauda epididymis of adult *Kif2c* HET and KO mice. (F) The number of mature spermatozoa obtained from *Kif2c* HET and KO cauda epididymis. ***p < 0.001, Student's *t*‐test.

To understand the cause of male infertility, we analyzed the testes of *Kif2c* KO mice. The gross morphology of the testis was obviously smaller in *Kif2c* KO mice than that in *Kif2c* HET mice (Figure 3A). In addition, while there were no significant differences in the body weight between *Kif2c* HET and KO mice (Figure 3B), the ratio of testis weight to body weight was significantly lower in *Kif2c* KO mice compared to *Kif2c* HET mice (Figure 3C).

To further investigate the cause of small testes, we performed histological analyses of testicular sections from adult *Kif2c* HET and KO mice (Figure 3D). PAS staining of testicular sections from *Kif2c* HET mice showed normal spermatogenesis. In contrast, the lumens of the seminiferous tubules in *Kif2c* KO mice were filled with vacuoles, indicating severe spermatogenesis defects. Furthermore, no mature spermatozoa were present in the cauda epididymis of *Kif2c* KO mice, while numerous spermatozoa were found in that of *Kif2c* HET mice (Figure 3E). We also counted the number of spermatozoa collected from the cauda epididymis and found no spermatozoa in *Kif2c KO* mice (Figure 3F). These results indicate that *Kif2c* deletion leads to nonobstructive azoospermia associated with hypospermatogenesis.

### 3.5 KIF2C Plays a Crucial Role in Meiosis During Spermatogenesis

*Kif2c* KO mice exhibit nonobstructive azoospermia; however, the severity of hypospermatogenesis remains unclear. Mouse spermatogenesis progresses from spermatogonial stem cells through meiosis I and II, ultimately producing haploid spermatocytes [33]. Following the second meiotic division, round spermatids undergo drastic morphological changes called spermiogenesis, transforming into elongated spermatids and spermatozoa [34].

**FIGURE 4:**
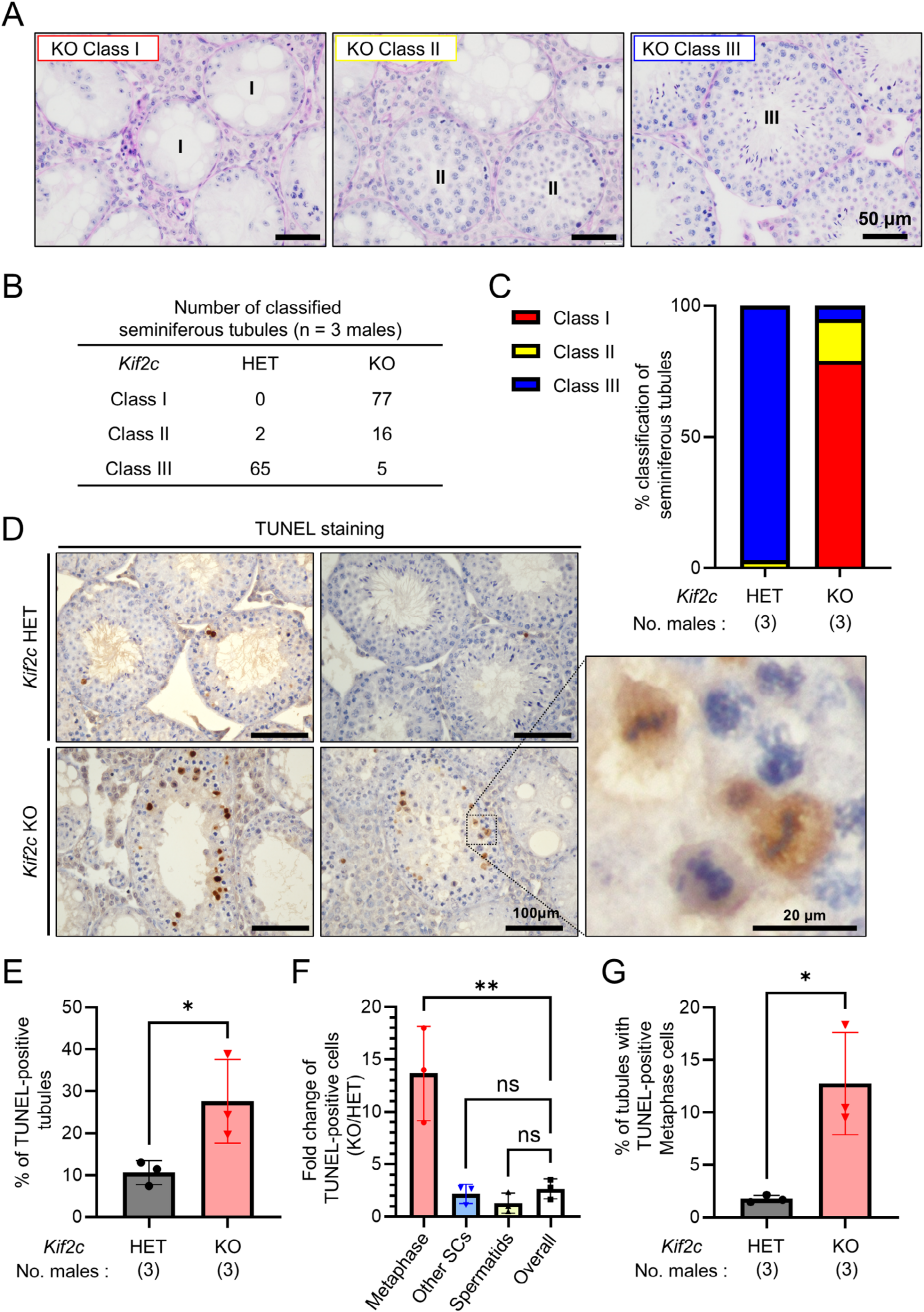
*Kif2c* KO male mice exhibited meiotic arrest at metaphase cells. (A) PAS staining of *Kif2c* KO seminiferous tubules. Seminiferous tubules were classified into three classes. The seminiferous tubules with only spermatogonia and/or spermatocytes were classified as Class I. Class II seminiferous tubules contain round spermatids but no elongating spermatids. Class III seminiferous tubules contain elongating spermatids. (B, C) The number of Class I‐III seminiferous tubules was counted in *Kif2c* HET and KO mice. (D) The TUNEL staining of *Kif2c* HET KO testes. Apoptotic cells were stained brown. (E) The percentage of seminiferous tubules containing apoptotic cells was compared between *Kif2c* HET and KO testes. *p < 0.05, Student's *t*‐test. (F) TUNEL‐positive cells in *Kif2c* HET and KO seminiferous tubules were categorized into three groups: Spermatocytes at metaphase, other spermatocytes (SCs), and spermatids. The number of TUNEL‐positive cells in KO divided by the number of TUNEL‐positive cells in HET for each category was calculated. *n* = 3 males, **p < 0.01, One‐way ANOVA followed by Dunnett's multiple comparison test. (G) The percentage of seminiferous tubules containing TUNEL‐positive spermatocytes at metaphase was compared between *Kif2c* HET and KO mice. *p < 0.05, Student's *t*‐test.

To assess the severity of hypospermatogenesis in *Kif2c KO* mice, we classified the seminiferous tubules into three categories (Class I to Class III) based on the progression of spermatogenesis as depicted in Figure 4A. Seminiferous tubules were categorized as Class I when only spermatogonia and/or spermatocytes were present. Class II tubules contained round but no elongating spermatids, while Class III tubules contained elongating spermatids. In *Kif2c HET* mice, almost all seminiferous tubules contained elongating spermatids, with 97% classified as Class III (Figure 4B,C). In contrast, in *Kif2c KO* mice, 79% of seminiferous tubules were classified as Class I without spermatids in the tubules. In addition, 16% were classified as Class II with round but no elongating spermatids in the tubules, and 5% of the seminiferous tubules were classified as Class III, containing elongating spermatids (Figure 4B,C). These results indicate that spermatogenesis was disrupted before the formation of round spermatids in most of the *Kif2c* KO seminiferous tubules.

We also performed TUNEL staining on testicular sections to analyze apoptosis (Figure 4D). We found a significantly higher percentage of TUNEL‐positive seminiferous tubules in *Kif2c* KO mice compared to *Kif2c* HET mice (Figure 4E). Furthermore, by investigating the spermatogenic stages of apoptotic cells, we found that apoptosis often occurs during the meiotic metaphase, where the chromosomes are aligned at the center of spermatocytes, in *Kif2c* KO testes (Figure 4F,G).

## 4 Discussion

In this study, we generated global *Kif2c* KO mice by crossing B6D2 mice with the 129 strain and overcame preweaning lethality associated with *Kif2c* deletion. The successful generation of the global *Kif2c* KO mouse model enabled us to investigate the roles of KIF2C in spermatogenesis and male fertility. Our results demonstrated that *Kif2c* KO male mice exhibit meiotic arrest and severe hypospermatogenesis, resulting in nonobstructive azoospermia (NOA). These findings provide evidence that KIF2C is essential for spermatogenesis and male fertility in mice.

Although cKO mice are often used to overcome lethality, CRE‐dependent recombination is not 100% efficient in the cKO approach [19, 20], which can make the interpretation of results difficult. In contrast, our strategy using mixed genetic backgrounds allows us to analyze the effects of gene deletion in all cells throughout the body. However, we should be aware of the potential influence of mixed genetic backgrounds on phenotypes [35]. For *Kif2c* deletion, the testicular phenotypes of our *Kif2c* KO mice are consistent with those of *Kif2c* germ cell‐specific cKO mice [18], indicating that the mixed genetic background does not mitigate the spermatogenetic phenotypes in contrast to the lethality. These phenotypic similarities also suggest that the impaired spermatogenesis observed in our global *Kif2c* KO mice is likely attributable to the loss of *Kif2c* in germ cells.

Our histological analysis revealed that the majority of seminiferous tubules were arrested at the early stages of spermatogenesis, particularly during meiosis, with only a small proportion progressing to the stages of round and elongating spermatids. These results suggest that KIF2C is important for the normal completion of meiosis in mouse spermatocytes. In mouse spermatocytes, KIF2C co‐localizes with Aurora B, a key protein in chromosome segregation, in the inner domain of kinetochores in metaphases I and II [17], and it has been suggested that KIF2C ensures proper spindle attachment, corrects erroneous microtubule‐kinetochore interactions, and contributes to spindle formation near the centrosome [36, 37, 38]. These KIF2C functions are consistent with the meiotic arrest phenotypes observed in our *Kif2c* KO mice.

While KIF2C plays important roles in meiosis, some spermatogenic cells go through meiosis to become round and elongating spermatids without KIF2C. KIF2A, a paralog of KIF2C, may compensate for the KIF2C functions as *Kif2a* is also expressed in the early stages of spermatogenesis (Figure 1E). Although *Kif2a* global KO mice showed neonatal lethality [39], *Kif2a* knockdown in mouse oocytes resulted in metaphase arrest [40, 41], suggesting that KIF2A function may be similar to that of KIF2C. In contrast, *Kif2b* is highly expressed in spermatids but not in the early stages of spermatogenesis (Figure 1E), and its global KO male mice were fertile [42]. Further analyses are needed to understand the functional differences among KIF2A, 2B, and 2C in mitosis and meiosis.

In conclusion, using global *Kif2c* KO mice, we reveal that *Kif2c* deletion results in NOA, which is one of the most severe causes of male infertility without curative treatment available [43, 44]. Global *Kif2c* KO mice generated in this study may provide a model to study meiotic abnormalities and male infertility, which may lead to a better understanding of the etiology of NOA and the development of new therapeutic strategies.

## Ethics Statement

All animal procedures were approved by the Institutional Animal Care and Use Committee at Research Institute for Microbial Diseases, The University of Osaka.

## Consent

The authors have nothing to report.

## Conflicts of Interest

The authors declare no conflicts of interest.

## Supporting information


**Data S1.** **Figure S1.** Comparison of KIF2C amino acid sequences between humans and mice.
**Table S1.** Primers and gRNAs used in this study.

## Data Availability

The data that support the findings of this study are available from the corresponding author upon reasonable request.

## References

[1] N. Hirokawa , Y. Noda , Y. Tanaka , and S. Niwa , “Kinesin Superfamily Motor Proteins and Intracellular Transport,” Nature Reviews. Molecular Cell Biology 10, no. 10 (2009): 682–696. 19773780 10.1038/nrm2774

[2] J. G. Ferreira , A. L. Pereira , and H. Maiato , “Microtubule Plus‐End Tracking Proteins and Their Roles in Cell Division,” International Review of Cell and Molecular Biology 309 (2014): 59–140. 24529722 10.1016/B978-0-12-800255-1.00002-8

[3] N. Hirokawa and Y. Noda , “Intracellular Transport and Kinesin Superfamily Proteins, KIFs: Structure, Function, and Dynamics,” Physiological Reviews 88, no. 3 (2008): 1089–1118. 18626067 10.1152/physrev.00023.2007

[4] S. F. Bakhoum , S. L. Thompson , A. L. Manning , and D. A. Compton , “Genome Stability Is Ensured by Temporal Control of Kinetochore‐Microtubule Dynamics,” Nature Cell Biology 11, no. 1 (2009): 27–35. 19060894 10.1038/ncb1809 PMC2614462

[5] A. L. Manning , N. J. Ganem , S. F. Bakhoum , M. Wagenbach , L. Wordeman , and D. A. Compton , “The Kinesin‐13 Proteins Kif2a, Kif2b, and Kif2c/MCAK Have Distinct Roles During Mitosis in Human Cells,” Molecular Biology of the Cell 18, no. 8 (2007): 2970–2979. 17538014 10.1091/mbc.E07-02-0110 PMC1949365

[6] S. Liu , Z. Ye , V. W. Xue , Q. Sun , H. Li , and D. Lu , “KIF2C Is a Prognostic Biomarker Associated With Immune Cell Infiltration in Breast Cancer,” BMC Cancer 23, no. 1 (2023): 307. 37016301 10.1186/s12885-023-10788-4 PMC10071625

[7] J. Guo , W. Zhang , L. Sun , et al., “KIF2C Accelerates the Development of Non‐Small Cell Lung Cancer and Is Suppressed by miR‐186‐3p via the AKT‐GSK3β‐β‐Catenin Pathway,” Scientific Reports 13, no. 1 (2023): 7288. 37142638 10.1038/s41598-023-30073-5 PMC10160078

[8] B. Zhang , P. Liu , Y. Li , et al., “Multi‐Omics Analysis of Kinesin Family Member 2C in Human Tumors: Novel Prognostic Biomarker and Tumor Microenvironment Regulator,” American Journal of Cancer Research 12, no. 11 (2022): 4954–4976. 36504885 PMC9729912

[9] P. Zhang , H. Gao , C. Ye , et al., “Large‐Scale Transcriptome Data Analysis Identifies KIF2C as a Potential Therapeutic Target Associated With Immune Infiltration in Prostate Cancer,” Frontiers in Immunology 13 (2022): 905259. 35720323 10.3389/fimmu.2022.905259 PMC9203693

[10] X. Zhang , Y. Li , P. Hu , L. Xu , and H. Qiu , “KIF2C Is a Biomarker Correlated With Prognosis and Immunosuppressive Microenvironment in Human Tumors,” Frontiers in Genetics 13 (2022): 891408. 35685442 10.3389/fgene.2022.891408 PMC9171145

[11] R. Q. Li , Y. Yang , L. Qiao , L. Yang , D. D. Shen , and X. J. Zhao , “KIF2C: An Important Factor Involved in Signaling Pathways, Immune Infiltration, and DNA Damage Repair in Tumorigenesis,” Biomedicine & Pharmacotherapy 171 (2024): 116173. 38237349 10.1016/j.biopha.2024.116173

[12] Z. Xu , R. Miao , T. Han , et al., “KIF2C as a Potential Therapeutic Target: Insights From Lung Adenocarcinoma Subtype Classification and Functional Experiments,” Molecular Omics 20, no. 6 (2024): 417–429. 38940931 10.1039/d4mo00044g

[13] H. Cao , Z. Wan , F. Wang , Z. Liu , X. Li , and J. Hou , “Downregulation of KIF2C and TEKT2 Is Associated With Male Infertility and Testicular Carcinoma,” Aging (Albany NY) 13, no. 19 (2021): 22898–22911. 34591790 10.18632/aging.203583 PMC8544317

[14] H. He , T. Huang , F. Yu , et al., “KIF2C Affects Sperm Cell Differentiation in Patients With Klinefelter Syndrome, as Revealed by RNA‐Seq and scRNA‐Seq Data,” FEBS Open Bio 12, no. 8 (2022): 1465–1474. 10.1002/2211-5463.13446 PMC9340869 35622500

[15] A. Bojesen and C. H. Gravholt , “Klinefelter Syndrome in Clinical Practice,” Nature Clinical Practice. Urology 4 (2007): 192–204. 17415352 10.1038/ncpuro0775

[16] A. Bojesen , S. Juul , and C. H. Gravholt , “Prenatal and Postnatal Prevalence of Klinefelter Syndrome: A National Registry Study,” Journal of Clinical Endocrinology and Metabolism 88 (2003): 622–626. 12574191 10.1210/jc.2002-021491

[17] M. T. Parra , R. Gómez , A. Viera , et al., “A Perikinetochoric Ring Defined by MCAK and Aurora‐B as a Novel Centromere Domain,” PLoS Genetics 2, no. 6 (2006): e84. 16741559 10.1371/journal.pgen.0020084 PMC1472701

[18] R. Harima , M. Kishinami , K. Hara , and K. Tanemura , “KIF2C Is Essential for Meiosis and Manchette Dynamics in Male Mice,” Frontiers in Cell and Development Biology 13 (2025): s1523593. 10.3389/fcell.2025.1523593 PMC11983436 40213390

[19] P. I. Sadate‐Ngatchou , C. J. Payne , A. T. Dearth , and R. E. Braun , “Cre Recombinase Activity Specific to Postnatal, Premeiotic Male Germ Cells in Transgenic Mice,” Genesis 46, no. 12 (2008): 738–742. 18850594 10.1002/dvg.20437 PMC2837914

[20] K. Tokuhiro , Y. Satouh , K. Nozawa , et al., “Calreticulin Is Required for Development of the Cumulus Oocyte Complex and Female Fertility,” Scientific Reports 5 (2015): 14254. 26388295 10.1038/srep14254 PMC4585710

[21] “International Mouse Phenotyping Consortium,” accessed February 20, 2025, https://www.mousephenotype.org/data/supporting‐data?mgiGeneAccessionId=MGI:1921054&mpTermId=MP:0011100.

[22] M. E. Dickinson , A. M. Flenniken , X. Ji , et al., “High‐Throughput Discovery of Novel Developmental Phenotypes,” Nature 537, no. 7621 (2016): 508–514. 27626380 10.1038/nature19356 PMC5295821

[23] B. P. Hermann , K. Cheng , A. Singh , et al., “The Mammalian Spermatogenesis Single‐Cell Transcriptome, From Spermatogonial Stem Cells to Spermatids,” Cell Reports 25, no. 6 (2018): 1650–1667. 30404016 10.1016/j.celrep.2018.10.026 PMC6384825

[24] F. Madeira , Y. M. Park , J. Lee , et al., “The EMBL‐EBI Search and Sequence Analysis Tools APIs in 2019,” Nucleic Acids Research 47, no. W1 (2019): W636–W641. 30976793 10.1093/nar/gkz268 PMC6602479

[25] A. M. Waterhouse , J. B. Procter , D. M. Martin , M. Clamp , and G. J. Barton , “Jalview Version 2—A Multiple Sequence Alignment Editor and Analysis Workbench,” Bioinformatics 25, no. 9 (2009): 1189–1191. 19151095 10.1093/bioinformatics/btp033 PMC2672624

[26] F. Abbasi , H. Miyata , K. Shimada , et al., “RSPH6A Is Required for Sperm Flagellum Formation and Male Fertility in Mice,” Journal of Cell Science 131, no. 19 (2018): 221648, 10.1242/jcs.221648. PMC6198453 30185526

[27] Y. Ho , K. Wigglesworth , J. J. Eppig , and R. M. Schultz , “Preimplantation Development of Mouse Embryos in KSOM: Augmentation by Amino Acids and Analysis of Gene Expression,” Molecular Reproduction and Development 41 (1995): 232–238. 7654376 10.1002/mrd.1080410214

[28] Y. Muro , H. Hasuwa , A. Isotani , et al., “Behavior of Mouse Spermatozoa in the Female Reproductive Tract From Soon After Mating to the Beginning of Fertilization,” Biology of Reproduction 94, no. 4 (2016): 80. 26962112 10.1095/biolreprod.115.135368

[29] A. Morohoshi , H. Miyata , K. Shimada , et al., “Nexin‐Dynein Regulatory Complex Component DRC7 but Not FBXL13 Is Required for Sperm Flagellum Formation and Male Fertility in Mice,” PLoS Genetics 16, no. 1 (2020): e1008585. 31961863 10.1371/journal.pgen.1008585 PMC6994161

[30] H. Miyata , A. Morohoshi , and M. Ikawa , “Analysis of the Sperm Flagellar Axoneme Using Gene‐Modified Mice,” Experimental Animals 69, no. 4 (2020): 374–381. 32554934 10.1538/expanim.20-0064 PMC7677079

[31] M. C. Lemos , B. Harding , A. A. Reed , et al., “Genetic Background Influences Embryonic Lethality and the Occurrence of Neural Tube Defects in Men1 Null Mice: Relevance to Genetic Modifiers,” Journal of Endocrinology 203, no. 1 (2009): 133–142. 19587266 10.1677/JOE-09-0124

[32] S. Shimada , T. Yoshizawa , Y. Takahashi , et al., “Backcrossing to an Appropriate Genetic Background Improves the Birth Rate of Carbohydrate Sulfotransferase 14 Gene‐Deleted Mice,” Experimental Animals 69, no. 4 (2020): 407–413. 32522905 10.1538/expanim.19-0150 PMC7677086

[33] L. D. Russell , R. A. Ettlin , A. P. Sinha Hikim , and E. D. Clegg , Histological and Histopathological Evaluation of the Testis (Cache River Press, 1990), 41–58.

[34] H. Miyata , K. Shimada , Y. Kaneda , and M. Ikawa , “Development of Functional Spermatozoa in Mammalian Spermiogenesis,” Development 151, no. 14 (2024): dev202838, 10.1242/dev.202838. 39036999

[35] R. S. Sellers , “The Gene or Not the Gene That Is the Question: Understanding the Genetically Engineered Mouse Phenotype,” Veterinary Pathology 49, no. 1 (2012): 5–15. 21971987 10.1177/0300985811421324

[36] H. Huang , J. Feng , J. Famulski , et al., “Tripin/hSgo2 Recruits MCAK to the Inner Centromere to Correct Defective Kinetochore Attachments,” Journal of Cell Biology 177, no. 3 (2007): 413–424. 17485487 10.1083/jcb.200701122 PMC2064832

[37] S. B. Domnitz , M. Wagenbach , J. Decarreau , and L. Wordeman , “MCAK Activity at Microtubule Tips Regulates Spindle Microtubule Length to Promote Robust Kinetochore Attachment,” Journal of Cell Biology 197, no. 2 (2012): 231–237. 22492725 10.1083/jcb.201108147 PMC3328376

[38] K. Tanaka , “Dynamic Regulation of Kinetochore‐Microtubule Interaction During Mitosis,” Journal of Biochemistry 152, no. 5 (2012): 415–424. 22995986 10.1093/jb/mvs109

[39] N. Homma , Y. Takei , Y. Tanaka , et al., “Kinesin Superfamily Protein 2A (KIF2A) Functions in Suppression of Collateral Branch Extension,” Cell 114, no. 2 (2003): 229–239. 12887924 10.1016/s0092-8674(03)00522-1

[40] M. H. Chen , Y. Liu , Y. L. Wang , et al., “KIF2A Regulates the Spindle Assembly and the Metaphase I–Anaphase I Transition in Mouse Oocyte,” Scientific Reports 6 (2016): 39337. 27991556 10.1038/srep39337 PMC5171862

[41] Z. Y. Yi , X. S. Ma , Q. X. Liang , et al., “Kif2a Regulates Spindle Organization and Cell Cycle Progression in Meiotic Oocytes,” Scientific Reports 6 (2016): 38574. 27991495 10.1038/srep38574 PMC5171826

[42] H. Miyata , J. M. Castaneda , Y. Fujihara , et al., “Genome Engineering Uncovers 54 Evolutionarily Conserved and Testis‐Enriched Genes That Are Not Required for Male Fertility in Mice,” Proceedings of the National Academy of Sciences of the United States of America 113, no. 28 (2016): 7704–7710. 27357688 10.1073/pnas.1608458113 PMC4948324

[43] L. Kasak and M. Laan , “Monogenic Causes of Non‐Obstructive Azoospermia: Challenges, Established Knowledge, Limitations and Perspectives,” Human Genetics 140, no. 1 (2021): 135–154. 31955275 10.1007/s00439-020-02112-y

[44] K. Chiba , N. Enatsu , and M. Fujisawa , “Management of Non‐Obstructive Azoospermia,” Reproductive Medicine and Biology 15, no. 3 (2016): 165–173. 29259433 10.1007/s12522-016-0234-z PMC5715857

