# Supplementary material for: KIF2C Deletion Causes Meiotic Abnormalities and Nonobstructive Azoospermia in Mice
Source: Reprod Med Biol. 2025 May 27;24(1):e12659. doi: 10.1002/rmb2.12659 (PMC12107603; doi:10.1002/rmb2.12659)
Supplement: Supplementary file 1 — Data S1. Figure S1. Comparison of KIF2C amino acid sequences between humans and mice. Table S1. Primers and gRNAs used in this study. [file RMB2-24-e12659-s001.pdf]

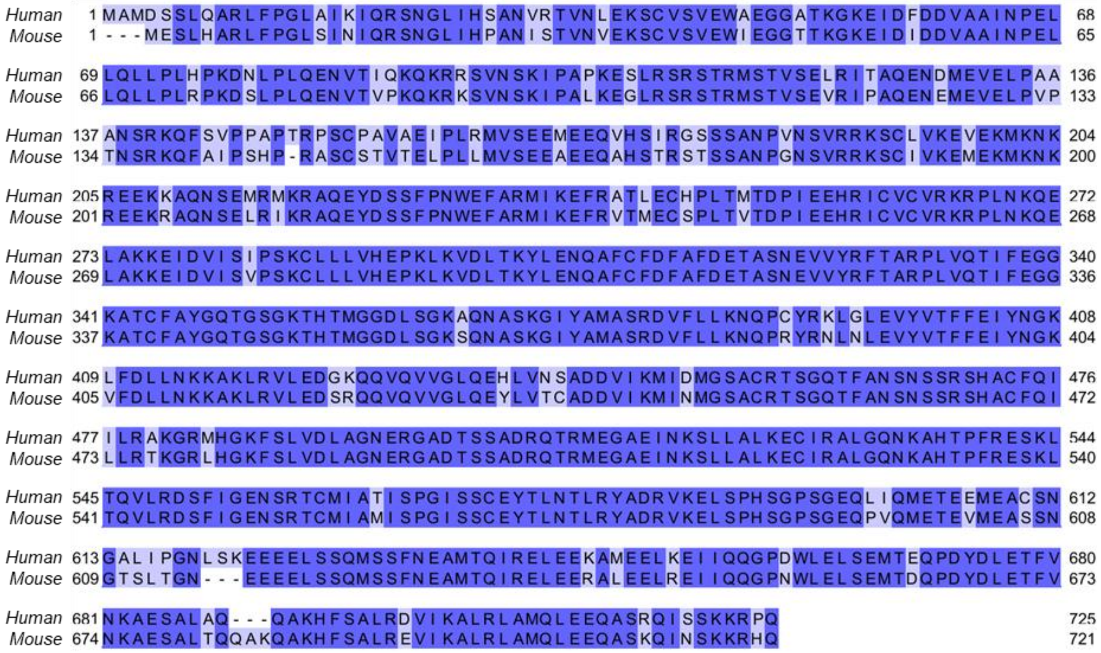

Supplementary Figure S1. Comparison of KIF2C amino acid sequences between humans and mice.

Alignment of the amino acid sequences of human and mouse KIF2C was performed using Clustal Omega and Jalview.

Supplementary Table S1. Primers and gRNAs used in this study.

primer	sequence (5'-3')
Kif2c Fw #1	gccataaaaccttcccatca
Kif2c Fw #2	cagaaacagcatgagcagga
Kif2c Rv #1	tggcaaatgtttgtccagaa
Kif2c Rv #2	ctgtccctggactcagaagg
Kif2c Fw for RT-PCR	aagaattcgccgccatggagtcgcttcacgcacgcc
Kif2c Rv for RT-PCR	aagatatcttggtgccgtttcttgctg
Actb Fw for RT-PCR	catccgtaaagacctctatgccaac
Actb Rv for RT-PCR	atggagccaccgatccaca

gRNA	target sequence (5'-3')
Kif2c gRNA #1	gcactggtgacgttgtaggg
Kif2c gRNA #2	ctggtggaaacctcgctgcg
